# Angle-independent VO_2_ Thin Film on Glass Fiber Cloth as a Soft-Smart-Mirror (SSM)

**DOI:** 10.1038/srep37264

**Published:** 2016-11-16

**Authors:** Nianjin Cai, Wang Zhang, Wanlin Wang, Yuchen Zhu, Imran Zada, Jiajun Gu, Qinglei Liu, Huilan Su, Cuiping Guo, Zhijian Zhang, Jianzhong Zhang, Liping Wu, Di Zhang

**Affiliations:** 1 State Key Laboratory of Metal Matrix Composites Shanghai Jiao Tong University, 800 Dongchuan Road, Shanghai, 200240, P.R. China; 2 College of Electronic Science and Technology, Shenzhen University, Shenzhen 518060, P.R. China; 3 Jushi Fiberglass Research Institute, Jushi Group Co., Ltd. 669 Wenhua Road(South), Tongxiang Economic Development Zone, Tongxiang City, Zhejiang Province 314500, P.R. China

## Abstract

Designing materials with a negative feedback function is beneficial for achieving temperature regulation inside a greenhouse. VO_2_ has been studied extensively because of its low insulator-to-metal transition temperature (IMT). In this study, reflection changes during a VO_2_ phase transition were investigated. Glass fiber cloth was used as a substrate, as it is stable and soft. A VO_2_ thin film on a glass fiber cloth whose surface contained 96% V^4+^ and 4% V^5+^ was prepared using an inorganic *sol-gels* method. The insulator-to-metal transition temperature was decreased by 38 °C, which was observed from the reflection curve detected using an angle-resolved spectrometer. This decrease in IMT occurred mainly because of the presence of V^5+^, which causes destabilization of the monoclinic phase of VO_2_. When the greenhouse temperature was increased from 30 °C to 40 °C, the reflected intensity of VO_2_ on glass fiber cloth decreased by 22% for the wavelength range of 400 nm to 800 nm. In addition, the angle-independent property of the VO_2_ thin film was observed using an angle-resolved spectrometer. Owing to its thermo-reflective properties, the thin film can serve as a soft-smart-mirror (SSM) inside a greenhouse to stabilize the temperature, playing a negative feedback role.

Plants can grow rapidly in greenhouses because of the proper CO_2_ concentration, humidity and temperature in this environment. However, light sources inside the greenhouse, which are mainly present to provide light for the photosynthesis needs of plants, can increase the temperature. Thus, stabilizing the temperature inside the greenhouse is necessary for plant growth. To regulate the temperature, designing a material with a negative feedback function is promising. The negative feedback function works as follows: when the temperature is lower than the target, the material will provide heat, resulting in an increase in temperature. In contrast, when the temperature is higher than the target, the material will absorb heat, resulting in a decrease in temperature. To achieve this negative feedback function, a VO_2_ thin film on a glass fiber cloth was fabricated in this study.

VO_2_ was first discovered to undergo a first-order phase transition from a monoclinic (P21/c) to a tetragonal (rutile—P42/mnm) crystalline structure at an insulator-to-metal transition temperature (IMT) of 68 °C in 1959^1^. During the phase transition, the conductivity, optical transmittance, permeability and specific heat capacity of VO_2_ can be altered in a short time^2^. Therefore, VO_2_ has potential use in thermoelectric^3^,^4^, thermomagnetic^5^,^6^,^7^ and thermo-optic materials^8^,^9^,^10^. The IMT of VO_2_ is the target temperature of the negative feedback system inside a greenhouse. However, this is typically much higher than room temperature and is not suitable for plant growth. To decrease the IMT of VO_2_, past studies have been investigated doping VO_2_ with W^11^,^12^,^13^,^14^, H^15^, F^13^,^16^ and Mg^17^,^18^,^19^. In this study, by adjusting the technological parameters of the experiment, the IMT of the VO_2_ thin film was further decreased to 38 °C, which resulted from incorporating a proper proportion of V^5+^. An IMT of 38 °C, after doping with V^5+^, as the target temperature in a negative feedback system inside a greenhouse is suitable for the growth of some plants. In this study, the negative feedback mechanism of VO_2_ was achieved owing to its thermo-optical properties.

The thermo-optical properties of VO_2_ thin films are a promising area of research. When the transition occurs, the index of refraction changes suddenly^20^, resulting in a sudden change in the transmittance and reflectance of VO_2_. In the monoclinic phase, the transmittance of infrared through VO_2_ is high, whereas in the other phases it is low. Based on this property, VO_2_ thin films have been studied for use as smart windows^8^,^10^,^19^,^21^. Another significant related area of research is altering the reflection of VO_2_ by changing the temperature. When temperature changes, the reflection changes correspondingly, making this material useful for optical temperature sensors^22^. The main focus of the present study is the reflection of the VO_2_ thin film surface during temperature changes. This thermo-reflective property has great potential for regulating the temperature inside a greenhouse by providing negative feedback.

In this study, glass fiber cloth was used as the substrate of the VO_2_ thin film. The weaving structure of the glass fiber cloth was the result of the VO_2_ thin film angle independent from the reflection changing rule. Moreover, glass fiber is inert in a *sol-gel* solution and stable under heat treatment with a coating layer. In addition, glass fiber cloth is soft, and therefore can tolerate bending.

On the surface of the VO_2_ thin film, supported by the glass fiber cloth, a change in the reflected intensity of more than 22% in the wavelength range of 400 nm to 800 nm was observed during heating and cooling. The crystal structural conversion from monoclinic at low temperature to tetragonal rutile at high temperature is an essential feature of this phenomenon.

Based on the property of the coating layer and the characteristics of the glass fiber cloth, the novel concept of a soft-smart-mirror (SSM) is proposed. The SSM can be used inside a greenhouse to stabilize the temperature. The negative feedback mechanism of SSM is as follows. When the temperature is lower than the IMT, the reflection is high. As a result, the SSM turns on, and the temperature inside the greenhouse subsequently increases. When the temperature is higher than the IMT, the reflection decreases more than 30% from 650 nm to 800 nm. As a result, the SSM turns off, and the temperature inside the greenhouse subsequently decreases. In this way, the SSM plays an important role in temperature regulation. Moreover, when the temperature increases, the small decrease in reflection in the wavelength range of 400 nm to 650 nm is beneficial for plant growth through photosynthesis.

There are various methods of fabricating VO_2_ thin films, such as chemical vapor deposition (CVD)^9^,^23^,^24^, magnetron sputtering^2^,^19^,^21^,^25^,^26^, and *sol-gel* synthesis^27^,^28^,^29^. *Sol-gel* synthesis has been proven to be a convenient route to fabricate VO_2_ thin films. In the study presented here, VO_2_ thin films are synthesized using the sol-gel method, and their structure and optical properties are characterized.

## Results

### Surface morphologies

**Figure 1:**
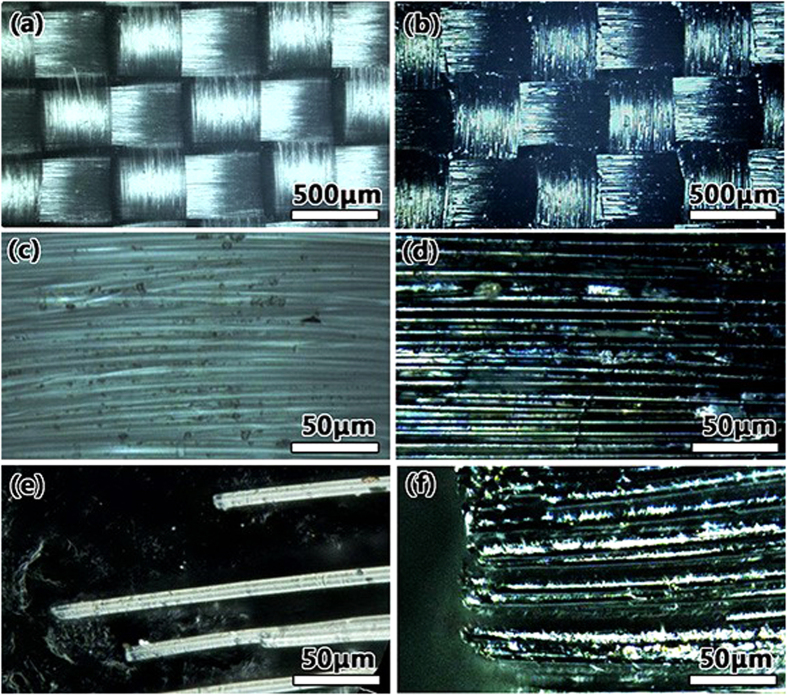
(**a**), (**b**) and (**e**) Original glass fiber cloth. (**b**) Morphologies of the glass fiber cloth coated with VO_2_. (**d**) Central region of the glass fiber cloth coated with VO_2_ (shown in light blue). (**f** ) Outcrop glass fiber coated with VO_2_. The coating is uniform.

Mesoscopic images of the surface morphologies from VHX are shown in Fig. 1. The light source of VHX was a halogen lamp (12 V 100 W), with a wavelength ranging from 360 nm to 2500 nm. The wavelength band covers the full spectrum of visible light. Figure 1(a,c,e) show white views of the original glass fiber cloth with different magnifications. Perpendicular bunches of glass fibers were woven into cloth, and every bunch consisted of multiple glass fibers (Fig. 1(a)). Figure 1(c) shows that the diameter of the glass fiber was homogeneous. An outcrop of the glass fiber is shown in Fig. 1(e). Figure 1(b,d,f) show the glass fiber cloth coated with the VO_2_ thin film at different magnifications. The texture and size of the glass fiber cloth remained the same, which indicated that the *sol-gel* synthesis method did not change the texture of the cloth or its size. It also indicated that the thickness of the coating layer was very thin; otherwise, the weaving structure would disappear, as shown in Fig. 1(d). By comparing Fig. 1(c) with (d), we find that the texture of both glass fibers is clearly visible and that the coating layer fills the gap between adjacent glass fibers. Moreover, the outcrop of glass fiber cloth was coated well after dipping-baking (Fig. 1(f)).

**Figure 2:**
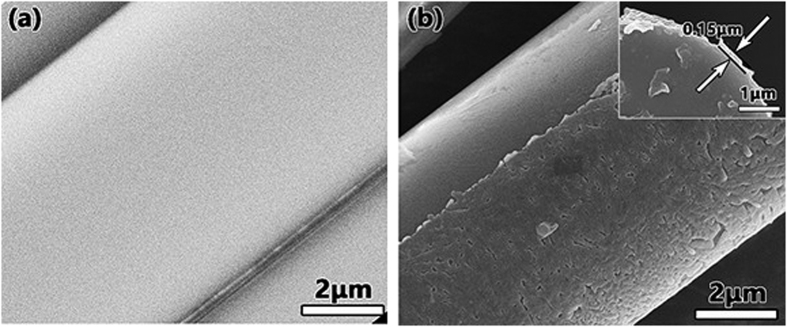
(**a**) Original glass fiber. (**b**) Glass fiber with a coating layer. Inset of (**b**): the cross-section of a glass fiber with a coating layer.

The microstructure images from SEM are shown in Fig. 2. Figure 2(a,b) show images of the original glass fiber and a glass fiber coated with VO_2_ thin film, respectively. Figure 2(a,b) show that the diameter of the original glass fiber was 7.15 ± 0.02 μm and that the thickness of the coated layer was 0.17 ± 0.04 μm. The inset of Fig. 2(b) shows a cross-section of a glass fiber with a VO_2_ thin film, from which we measured the thickness of the coating layer to be approximately 0.15 μm. As for the thickness of the coating layer, the initial experiment shows that different technological parameters determine whether the VO_2_ crystal was generated and the proportion of vanadium ions in different valence states, as shown in the Supporting Information.

### Constituent

**Figure 3:**
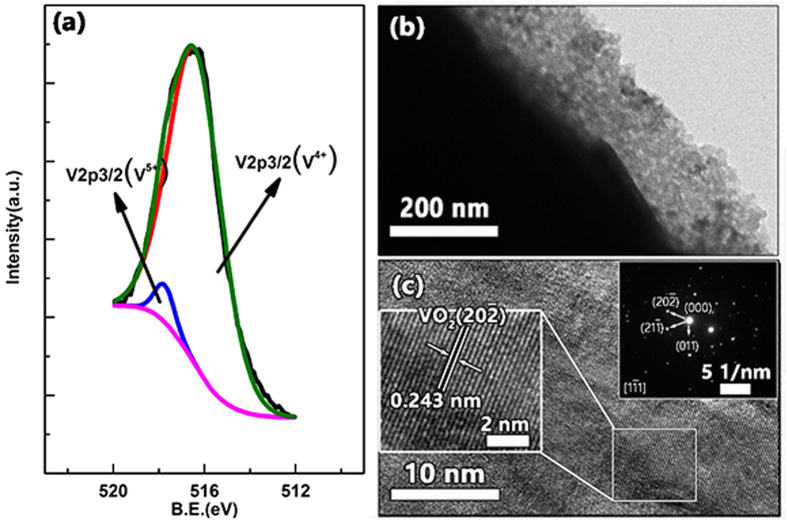
(**a**) Surface XP spectrum of the glass fiber cloth with a coating layer. (**b**) TEM image of a glass fiber with a coating layer. (**c**) The HRTEM image of the coating layer. The insets in (**c**) are the SAED image and HRTEM image.

A typical XP spectrum is shown in Fig. 3(a), in which the area below the peak represents the proportion of vanadium oxide. The spectrum shows that the proportions of V^4+^ and V^5+^ were approximately 96% and 4%, respectively.

TEM images are shown in Fig. 3(b,c). The average thickness of the coating layer on a glass fiber was from 130 nm to 200 nm (Fig. 3(b)), which matched the thickness observed from the SEM, as mentioned above (0.17 ± 0.04 μm). Figure 3(c) shows the HRTEM image of the coating layer. The insets of Fig. 3(c) are the SAED image and HRTEM image. The plane distances calculated from the SAED image of Fig. 3(c) are 0.243 nm, 0.243 nm and 0.320 nm, which fits well with the standard values of the VO_2_ crystal in the monoclinic phase (P21/c). The distances are in good agreement with the planes (hkl) = (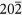) (d = 0.2433 nm), (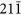) (d = 0.2428 nm) and (011) (d = 0.3200 nm), respectively. The HRTEM shown in the inset of Fig. 3(c) shows the plane (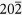) of a monoclinic VO_2_ crystal. Therefore, the structure of VO_2_ is monoclinic (P21/c) at a low temperature.

The doping of V^5+^ into the VO_2_ crystal at the proper proportion, as shown in Fig. 3(a), decreases the IMT to 38 °C. In a previous study, the formation of V^3+^ with tungsten doping caused a destabilization of the monoclinic phase of VO_2_ and a decrease in the insulator-to-metal transition temperature^30^,^31^. The existence of V^5+^ plays the same role as V^3+^ and decreases the IMT of the VO_2_ thin film.

### Thermo-reflective property

The thermo-reflective property is considered to be useful in regulating the temperature of a greenhouse. The light source inside the greenhouse not only provides necessary light for photosynthesis, but also increases the temperature. To ensure the rapid growth of plants, it is necessary to regulate the temperature of the greenhouse. When a thermo-reflection device with a negative feedback mechanism is placed inside a greenhouse, the temperature can be stabilized. The mechanism works as follows: the reflection of the device is low when the temperature is high, which will automatically decrease the temperature of the greenhouse. In contrast, when the temperature is low, the reflection will be high, which will increase the temperature inside the greenhouse.

**Figure 4:**
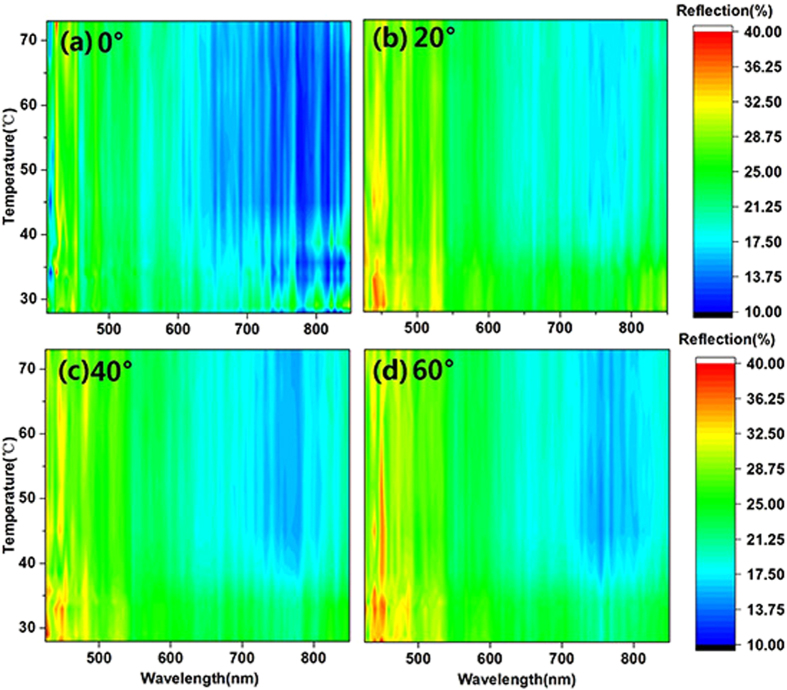
Contour map of the reflection of the glass fiber cloth coated with VO_2_ at different temperatures and different detection angles. (**a**), (**b**), (**c**) and (**d**) Contour map of the reflection of the glass fiber cloth coated with VO_2_ at detection angles 0°, 20°, 40° and 60°, respectively. The detected area is a circle region with a diameter of approximately 300 μm.

**Figure 5:**
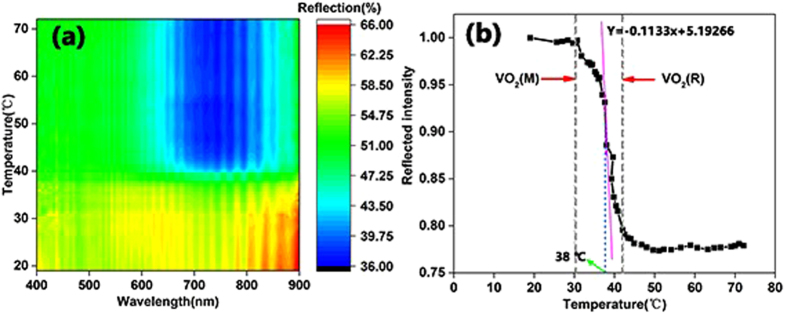
(**a**) Contour map of the total reflection from every detection angle of the glass fiber cloth coated with VO_2_. (**b**) Normalized thermo-reflected light intensity. The intensity was calculated by integrating the area below the wavelength-reflection curve at different temperatures. The area selected is a circle region with a diameter of approximately 30 μm.

In this work, the thermo-reflective property of a VO_2_ thin film on glass fiber cloth is observed using an angle-resolved spectrometer. The light source rays in the real application as well as the reflection of the device are always emitted in every direction. Thus, investigating the angle dependence of the VO_2_ thin film is absolutely necessary. The contour maps presented in Figs 4 and 5 are integrated from the reflection wavelength under different temperatures observed using an angle-resolved spectrometer with a heating device, whose temperature was monitored by a thermocouple. The light source was a halogen lamp (ARM.B-H100), with a wavelength range of 360 to 2500 nm and a light intensity distribution of mainly 400 to 800 nm, covering the visible light wavelength band. It is more intuitive to exhibit the relationships among reflection, wavelength and temperature in a contour map than other presentations.

Figure 4(a–d) are contour maps of the reflection of the glass fiber cloth coated with VO_2_ at detection angles of 0°, 20°, 40° and 60°, respectively. For these contour maps, the detection plane was perpendicular to the orientation of the glass fiber. Figure 5 (a) depicts the contour map of the total reflection from all angles of the glass fiber cloth coated with VO_2_. The diameters of the detected areas of Figs 4 and 5 are approximately 300 μm and 30 μm, respectively.

Figure 4 shows that, at different angles, the reflection of the VO_2_ coating on the glass fiber cloth has the same change rule. When the temperature reached 30 °C, the reflection of the VO_2_ surface decreased significantly. When the temperature reached 40 °C, the reflection became stable and was at a low level, especially at wavelengths of 650 nm to 850 nm. For wavelengths from 650 nm to 850 nm, the reflection decreased to 28%, 30%, 29% and 29% for detection angles of 0°, 20°, 40° and 60°, respectively. In addition, the reflection was almost stable from 400 nm to 600 nm when the temperature was changing. The contour maps show that the coating layer on the glass fiber has the properties of angle-independence and thermo-reflection. For further confirmation of the results, another experiment, for which the diameter of the detected area was approximately 1 mm, was performed using a macro angle-resolved spectrometer (R1 Ideaoptic). The area was larger than one period of the glass fiber cloth, which is roughly measured in Fig. 1(b). The results are shown in the support information Fig. S3(a–d) and were in good agreement with the results shown in Fig. 4.

VO_2_ thin films exhibit anisotropy in conductivity^32^,^33^ and index of refraction^34^, having different values in the directions of the c-axis and b-axis. The reflection is related to the index of refraction; thus, the VO_2_ thin film has an angle-dependent property. In this study, the curvature of the glass fiber surface resulted in diffuse reflection, which weakened the angle-dependent property of the VO_2_ thin film. Thus, the VO_2_ thin film on the glass fiber cloth possessed the angle-independent property. The angle-independent property of the VO_2_ thin film on the glass fiber cloth is beneficial for the stability of temperature in a greenhouse because this property can homogenously produce heat from reflected light.

From Fig. 5(a), the changing rule of total angle reflection is the same as the fixed angle reflection. Figure 5(b) shows that the thermo-reflective property was intuitive. When the temperature reached 40 °C, the reflected intensity of the coating layer of the glass fiber cloth decreased by 22%. A decrease in reflected intensity causes a decrease in the brightness of a surface. The fastest changing temperature was at 38 °C, shown in Fig. 5(b), which shows that the VO_2_ phase transition occurs at 38 °C. The decrease in IMT occurs because of the V^5+^ doping in the VO_2_ crystal and because there would be no other elements in the coated layer for the *sol-gel* synthesis, as shown in Fig. 3(a). The presence of V^5+^ plays the same role as V^3+^ and causes the decrease in the IMT of the VO_2_ thin film. Based on this study, it is very promising to adjust the proportion of V^5+^ in a VO_2_ thin film to regulate the IMT of VO_2_.

**Figure 6:**
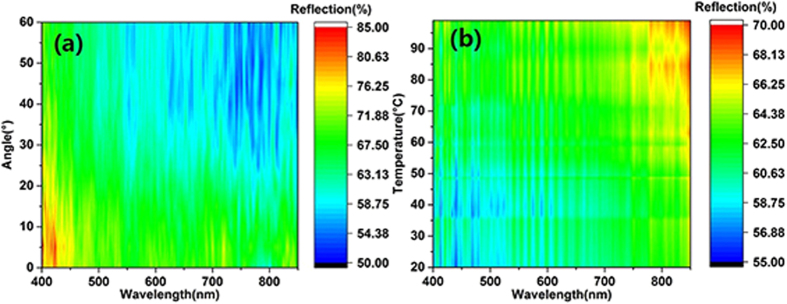
(**a**) Contour map of the reflection of the original glass fiber cloth for different detection angles. (**b**) Contour map of the reflection of the original glass fiber cloth at different temperatures.

To confirm that the glass fiber cloth has no influence on the thermo-reflective property, the reflections of the original glass fiber cloth with different temperatures or different detection angles were investigated (Fig. 6). Figure 6(a) shows that the reflection decreased with increasing detection angle. The reflection also decreased with increasing wavelength, which shows the angle-independent reflective property of the original glass fiber. Figure 6(b) shows that the reflection of the original glass fiber cloth decreased with increasing temperature, which is the opposite of the result observed for the glass fiber with coating layers, as shown in Fig. 5(a). Moreover, there no wavelength resolution was observed in the original glass fiber, but was observed in the glass fiber with the coating layer (Fig. 5(a)).

The reflection of the VO_2_ thin film on the glass fiber cloth at a wavelength range of 250 nm to 2500 nm at 20 °C, 30 °C, 35 °C, 45 °C, 60 °C and 75 °C was observed using a Lambda 750 S (PerkinElmer Co., Ltd), shown in the Supporting Information Fig. S3(e). The reflections at 45 °C, 60 °C and 75 °C were lower than those at 20 °C, 30 °C and 35 °C from 400 nm to 800 nm, which agrees well with the data from the angle-resolved spectrometer.

**Figure 7:**
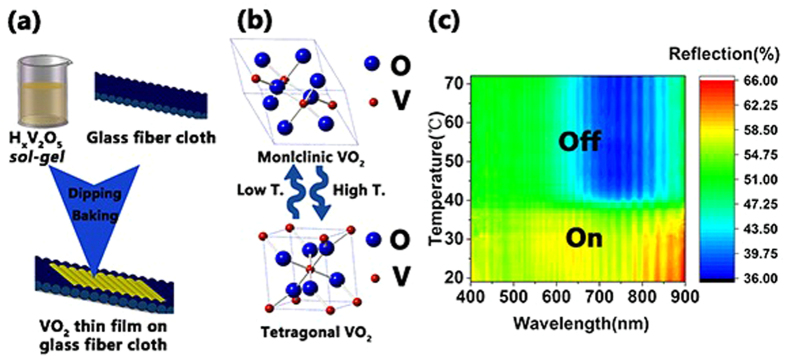
(**a**) VO_2_/GB soft-smart-mirror constructed by dipping-baking. The VO_2_ thin film supported by the glass fiber cloth. (**b**) Phase transition of VO_2_ for different temperatures. (**c**) Contour map of the reflection changing at different temperatures and different wavelengths.

The concept of a soft-smart-mirror (SSM) is shown in Fig. 7. Figure 7(a) shows that the *sol-gel* method fabricated a VO_2_ film supported by a glass fiber cloth. H_x_V_2_O_5_ *sol-gel* is the synthesis; thus, no other elements would appear in the thin film, ensuring the purity of the vanadium oxide thin film. The glass fiber cloth is used as a substrate not only because it is stable during dipping-baking but also because it is soft and therefore can tolerate bending. Figure 7(c) shows a contour map of the reflection changing with different temperatures and different wavelengths. Based on the property of VO_2_ thin films and the characteristics of the glass fiber cloth, the concept of the soft-smart-mirror (SSM) was proposed. When the temperature is lower than the IMT, the VO_2_ thin film stays in the monoclinic crystal structure, showing high reflection at 60%, and the SMM turns on. When temperature is higher than the IMT, the VO_2_ thin film stays in the tetragonal crystal structure, showing low reflection at 40%, and the SMM turns off. The crystal structural conversion from monoclinic at low temperature to tetragonal rutile at high temperature is an essential feature of this phenomenon, exhibited in Fig. 7(b). The SSM is very promising for use in greenhouses, playing the role of a temperature regulator inside the greenhouse. When the temperature inside the greenhouse is at a high level, the SSM is off, which results in a decrease in temperature and vice versa. Thus, the temperature inside the greenhouse would stabilize at an IMT of 38 °C, which is conducive to plant growth. Moreover, the SSM can serve as an optical temperature sensor. When the temperature is changing, the reflection will change correspondingly.

### Simulation

To further prove that the temperature of 38 °C is the IMT of the VO_2_ thin film doped with V^5+^, a simulation was performed. For this simulation, FDTD solution was used to analyze the reflection of the VO_2_ thin film as the temperature changed for different wavelength values.

**Figure 8:**
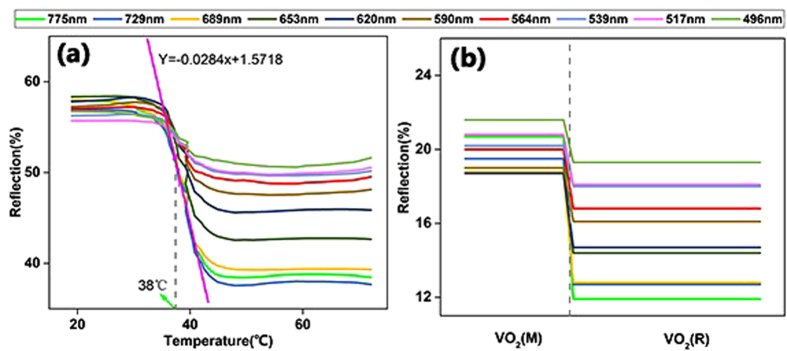
(**a**) The reflection of the VO_2_ thin film at different temperatures and 10 different wavelength values, observed using a spectrometer. (**b**) FDTD simulation result of the reflection of the VO_2_ thin film in different crystal structures and 10 different wavelength values.

Figure 8(a) shows 10 curves of the reflection from the experiment as the temperature changed for 10 different wavelength values. The FDTD simulation was performed for the 10 different wavelength values whose results of the reflection in different VO_2_ crystals are exhibited in Fig. 8(b). Comparison of the two graphs shown in Fig. 8 indicates that the reflection from the experiment was similar to the simulation. In the experiment, the reflection decreased sharply at 38 °C, which is the same outcome with the reflection changing rule in the simulation when the VO_2_ phase transition occurred. Detailed values of the decrease rate are shown in Table 1. Although the absolute values of reflection obtained from the experiment and simulation are different, the decrease rate calculated based on the reflection changing curve is similar. In fact, the absolute value of reflection varies with detection methods, but the decrease rate of reflection was not significantly different, irrespective of the detection method, because of the phase transition of VO_2_. In other words, from the simulation data, it could be concluded that 38 °C is the IMT of VO_2_ with V^5+^ doping in the VO_2_ crystal.

## Methods

### Pretreating for glass fiber cloth

The temperature of the muffle furnace was set at 500 °C for 2 hours with a heating rate at 2 °C/min. Then, the glass fiber cloth was placed in an ultrasonic machine for 1 hour to remove organic residue. Finally, the cloth was dried^35^.

### Synthesis of the VO_2_ thin film on the glass fiber cloth via the sol-gel method

Detailed information is reported in the Supporting Information.

### Characterization

The angle-independent thermo-reflective property of the VO_2_ thin film on the glass fiber cloth was observed using an angle-resolved spectrometer (ARM, Ideaoptics) with a heating device in the temperature range of 20 °C to 75 °C, monitored by a thermocouple. The reflection of the VO_2_ thin film on the glass fiber cloth in the temperature of 20 °C, 30 °C, 35 °C, 45 °C, 60 °C and 75 °C was observed using a Lambda 750 S, an ultraviolet and visible spectrophotometer.

### Simulation

The finite different time domain (FDTD) method was used. Simulations were performed under normal incident light with a plane wave light source of 10 different wavelength values. The boundary condition in the vertical direction is absorbing (perfectly matched layer, PML), whereas the boundary condition in the horizontal direction is periodic (periodic boundary condition, PBC). In this study, the refractive index (n) and wave vector (k) used in the simulation refer to Balberg, I. and S. Trokman^20^; the detailed values are mentioned in the support file, Table S1.

## Additional Information

**How to cite this article**: Cai, N. *et al*. Angle-independent VO_2_ Thin Film on Glass Fiber Cloth as a Soft-Smart-Mirror (SSM). *Sci. Rep.* **6**, 37264; doi: 10.1038/srep37264 (2016).

**Publisher’s note**: Springer Nature remains neutral with regard to jurisdictional claims in published maps and institutional affiliations.

## Supplementary Material

Supplementary Information

## Figures and Tables

**Table 1:** Reflection of VO_2_ at different temperatures with varying wavelengths from the experiment and simulation results.

Wavelength (nm)	Experiment			Simulation		
	Reflection		Decrease rate = (R(T < 30 °C)-R(T > 40 °C))/R(T < 30 °C)	Reflection		Decrease rate = (R(M)-R(R))/R(M)
	VO_2_(T < 30 °C)	VO_2_(T > 40 °C)		VO_2_(M)	VO_2_(R)	
775	58.00%	38.50%	33.60%	20.70%	11.90%	42.50%
729	56.80%	37.50%	34.00%	19.50%	12.70%	34.90%
689	58.00%	39.50%	31.90%	18.80%	12.80%	31.90%
653	56.80%	42.50%	25.20%	18.70%	14.40%	23.00%
620	58.00%	45.30%	21.90%	18.70%	14.70%	21.40%
590	57.50%	47.50%	17.40%	19.00%	16.10%	15.30%
564	57.20%	49.20%	14.00%	20.00%	16.80%	16.00%
539	56.80%	50.00%	12.00%	20.20%	18.00%	10.90%
517	55.80%	50.00%	10.40%	20.80%	18.10%	13.00%
496	57.00%	51.00%	10.50%	21.60%	19.30%	10.60%
