# Supplementary material for: Angle-independent VO2 Thin Film on Glass Fiber Cloth as a Soft-Smart-Mirror (SSM)
Source: Sci Rep. 2016 Nov 16;6:37264. doi: 10.1038/srep37264 (PMC5110969; doi:10.1038/srep37264)
Supplement: Supplementary Information [file srep37264-s1.pdf]

Angle-independent VO_2 Thin Film on Glass Fiber Cloth as a Soft-Smart-Mirror (SSM)

Nianjin Cai, Wang Zhang, Wanlin Wang, Yuchen Zhu, Imran Zada, Jiajun Gu, Qinglei Liu, Huilan Su, Cuiping Guo, Zhijian Zhang, Jianzhong Zhang, Liping Wu and Di Zhang**

Supporting Information

Experimental section:

1 Starting Materials

Vanadium pentoxide (V_2O_5) (Strem Chemicals 98%) was used as starting material for VO_2 synthesis, purchased from Aladdin chemical reagent Ltd. Glass fiber cloth ((GFC, E-glass fiber cloth, alkali free, 100g/m^2) was used for substrate of VO_2 thin film, purchased from China Jushi Ltd. Distilled water was used for all process.

2 $H_XV_2O_5$ *sol-gel* Synthesis

Firstly, 5g of V_2O_5 was melted at 800 ℃ air phenomenon in muffle for 30 minutes. And then melted V_2O_5 was poured into 250 ml of deionized water.[1] After stirring for 10 min, it was filtered and deep brownish sol was obtained. According to J. Livage[2] and Shi, Huang[3], the deep brownish sol was $H_XV_2O_5$ *sol-gel*s. The advantage of $H_XV_2O_5$ *sol-gel* was that it ensured the purity of vanadium oxide thin film because there was no element except V, H and O in the *sol-gel* solution.

3 Glass fiber cloth coating with Vanadium Oxide Synthesis

3.1 Pretreating for glass fiber cloth

The lubricant on glass fiber surface could affect *sol-gel*s particles coating for its non-infiltration, so it is necessary to clear them out. In order to get rid of them, the glass fiber cloth was put into muffle for heat treatment. Set the temperature of muffle at

500 ℃ for 2 hours, the heating rate at 8 ℃/min. Then put it into ultrasonic machine for 1 hours to clean organic residuum out. Finally, the cloth was dried. The heat treatment of 500 ℃ has minor effect on the strength of glass fiber cloth and it is more convenient as compare to acid leaching.

3.2 The dipping process of glass fiber cloth

During initial experiment of this study, the coating layer wouldn't grow VO_2 crystal as the coating layer was too thin after heat treatment. To control the thickness of coating layer, designing the dipping times for experiment is necessary. The increasing weight rate of glass fiber cloth after 5 and 10 times dipping is approximately (2.73±0.58)% and (5.01±0.98)%, respectively. Detail information could be seen in support file fig. S2. From the increasing weight of glass fiber cloth, the thickness of coating layer could be calculated. Simple mold was drawn which is shown in support file.

From the cross section mold of glass fiber, it is observed that the surface area is 1.57 times of the flat surface. From the weaving mold of glass fiber cloth, the area is observed to be double of flat surface. Thus the surface area of 100 g of glass fiber cloth is considered to be 6.28 m^2.

The relative molecular mass of HxV_2O_5 and VO_2 is about 162 $gmol^{-1}$ and 83 $gmol^{-1}$ respectively. Supposed the weight of $H_xV_2O_5$ on 100 g of glass fiber cloth is 5g after dipping 10 times. Finally the weight of the VO_2 on glass fiber cloth was:

$$\mathrm{m}(VO_2) = \left(\mathrm{m}(H_XV_2O_5) / \mathrm{M}(H_XV_2O_5)\right) \cdot 2 \cdot \mathrm{M}(VO_2) \approx 5.12\mathrm{g}$$

The density of VO_2 is 4.26 g/cm^3, so the thickness of VO_2 on glass fiber cloth surface is:

$$h(VO_2) = \frac{\frac{m(VO_2)}{\rho(VO_2)}}{S} \approx 0.19\mu m$$

0.19 μm of thickness is proper for VO_2 thin film. [4]

In this study, to make the VO_2 on glass fiber cloth surface homogeneous, glass fiber cloth is dipped 10 times. The detailed process is as follow: dipped the cloth for 10 seconds and dried the cloth in drying oven. This process was repeated for 10 times. Finally, deep yellow glass fiber cloth was obtained.

3.3 Heating treatment

The sample was placed into vacuum furnace (Carbolite, GHA 12/300), setting the temperature at 500 ℃ for 1.5 h and heating rate at 8 ℃ min^{-1}. After the heat treatment, the dark blue glass fiber cloth was obtained.

4 Characterization

The morphologies of glass fiber cloth coating with vanadium oxide were observed by VHX-1000E (Keyence), TEM (JEOL-2100F, Japan) and SEM (JSM-7500F, Japan; S-4800, Hitachi). Besides, the constituent of vanadium oxide was investigated by XPS (AXIS ULTRA DLD, Kratos) and SAED (JEOL-2100F, Japan). Furthermore, the thermo-reflection of VO_2 on glass fiber cloth was observed by angle-resolved spectrometer (ARM, Ideaoptics).

5 Finite Different Time Domain (FDTD) method

The FDTD model of VO_2 thin film on glass fiber cloth was designed based on experiments. Simulations were performed under normal incident light with the plane wave light source of 10 different wavelength values. The boundary condition in the vertical direction is absorbing (perfectly matched layer, PML), whereas the boundary

condition in the horizontal direction is periodic (periodic boundary condition, PBC). In this study, the refractive index (n) and wave vector (k) used in the simulation was refer to Balberg, I. and S. Trokman [5], the detail value are mentioned in table. S1.

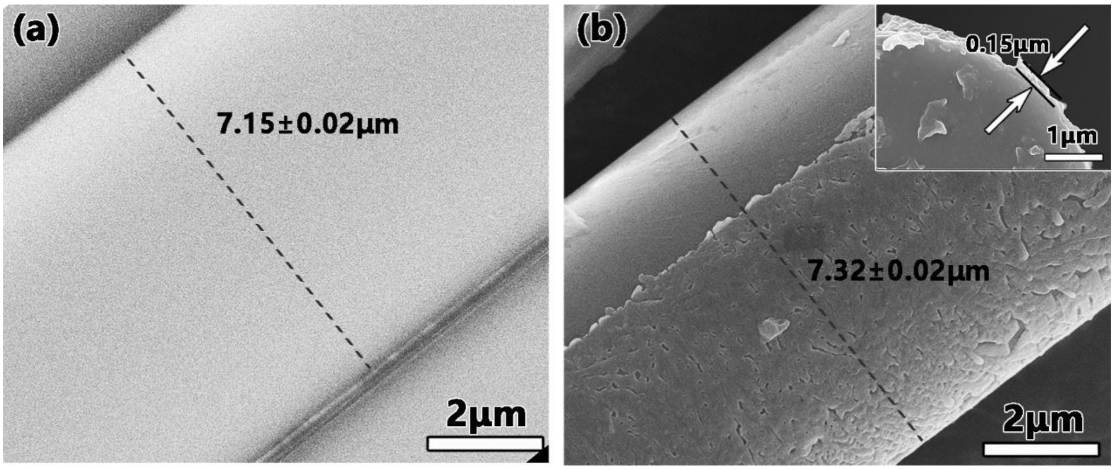

Fig. S1 (a) Original glass fiber. (b) Glass fiber with coated layer. Inset of (b): the cross section of glass fiber with coated layer.

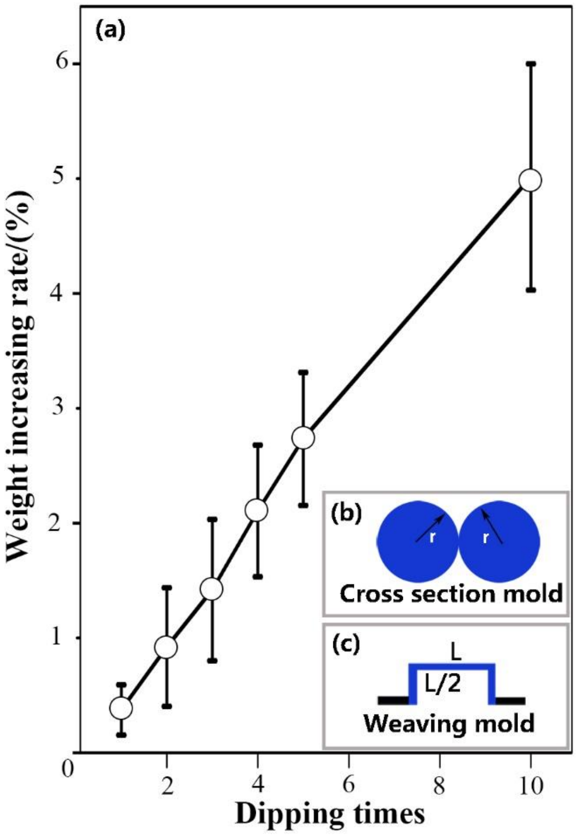

Fig. S2 (a) The increasing weight rate in different dipping times. (b) Cross section mold of glass fiber. (c) Weaving mold of glass fiber cloth.

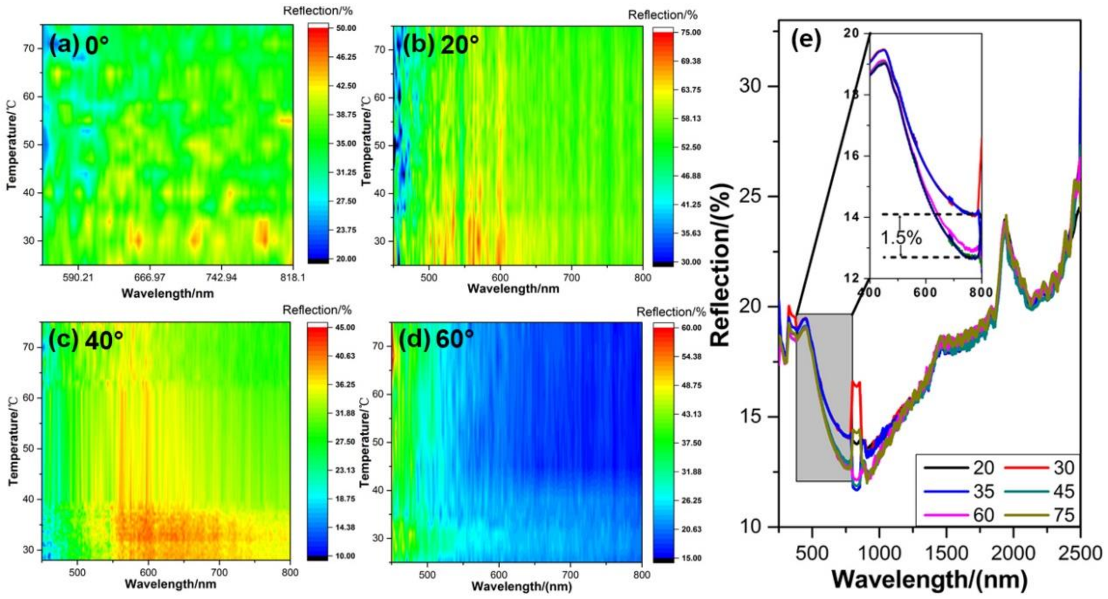

Fig. S3 (a)~(d)Contour map of reflection of glass fiber cloth coating with vanadium oxide for different temperature and different detection angle. (e) reflection of glass fiber cloth coating with vanadium oxide for different temperature in the wavelength range from 250 nm to 2500 nm. Insert: the amplify image of reflection in wavelength range from 400 nm to 800 nm. (a),(b),(c) and (d)Contour map of reflection of glass fiber cloth coating with vanadium dioxide in detection angle 0 °, 20 °, 40 °and 60 °, respectively.

Table. S1: The refractive index (n) and wave vector (k) in different wavelength and different crystal of VO_2 [5].

Wavelength/nm	E(eV)	VO2(M)		VO2(R)	
		n	k	n	k
775	1.6	2.81	0.94	1.67	0.90
729	1.7	2.76	0.78	1.92	0.83
689	1.8	2.74	0.69	2.04	0.74
653	1.9	2.77	0.60	2.19	0.78
620	2.0	2.81	0.53	2.25	0.75
590	2.1	2.86	0.45	2.38	0.76
564	2.2	2.97	0.44	2.45	0.78
539	2.3	2.98	0.44	2.5	0.88
517	2.4	3.05	0.44	2.49	0.90
496	2.5	3.11	0.50	2.53	1.01

References

1. Li, D., Huang, W., Song, L. & Shi, Q. Termal stability of VO_2 thin flms deposited by sol–gel method. *J. Sol-Gel Sci. Technol.* **75**,189–197 (2015).
2. Livage, J., Guzman, G., Beteille, F. & Davidson, P. Optical properties of sol-gel derived vanadium oxide flms. *J. Sol-Gel Sci. Technol.* **8**, 857–865 (1997).

3. Shi, Q. et al. Preparation and phase transition characterization of VO_2 thin flm on single crystal Si (100) substrate by sol–gel process.*J. Sol-Gel Sci. Technol.* **59,** 591–597 (2011).
4. Lu, S. W., Hou, L. S. & Gan, F. X. Structure and optical property changes of sol-gel derived VO_2 thin flms. *Adv. Mater.* **9**, 244-&(1997).
5. Balberg, I.; Trokman, S., High-contrast optical storage in VO_2 flms. *Jpn. J. Appl. Phys.* **46**, 2111 (1975)
